# Incorporation of oXiris Bioabsorbent Filter into CRRT in the treatment of severe abdominal infections and analysis of associated risk factors for early off-machine

**DOI:** 10.3389/fpubh.2025.1560587

**Published:** 2025-07-30

**Authors:** Yanfang Dong, Jieling Chen, Xiaoli Zhang, Shiyu Wu, Yiming Li, Qiaoxian Zhang

**Affiliations:** ^1^Department of Emergency Intensive Care Unit, The First Affiliated Hospital, Fujian Medical University, Fuzhou, China; ^2^Department of Nursing, The First Affiliated Hospital, Fujian Medical University, Fuzhou, China; ^3^Department of Trauma Center and Emergency Surgery, The First Affiliated Hospital, Fujian Medical University, Fuzhou, China; ^4^Department of Emergency, National Regional Medical Center, Binhai Campus of the First Affiliated Hospital, Fujian Medical University, Fuzhou, China

**Keywords:** early off-machine, risk factors, severe abdominal infections, oXiris Bioabsorbent Filter, CRRT

## Abstract

**Introduction:**

This study aims to evaluate the impact of oXiris continuous renal-replacement therapy (CRRT) on the prognosis of patients with severe intra-abdominal infections (IAIs) and to analyze potential risk factors for early off-machine of oXiris CRRT during treatment.

**Methods:**

A total of 49 patients with severe abdominal infections admitted to the intensive care unit of the First Affiliated Hospital of Fujian Medical University from October 2020 to October 2023 were retrospectively analyzed. The patients were divided into a conventional group and an oXiris group. Heart rate, blood lactate level, mean arterial pressure, and total CRRT operation time were observed 72 h before and after CCRT treatment.

**Results:**

When comparing changes in indicators over the 72-h period between the two groups, no significant difference in survival rate was observed between the two groups. D-dimer [per 1 ng/mL increase, odds ratio (OR) = 0.930, 95% confidence interval (CI): 0.866–0.999] was identified as a risk factor for early off-machine. In contrast, prothrombin time (PT, per 1-s increase, OR = 1.117, 95% CI: 1.017–1.226), activated partial thromboplastin time (APTT, per 1-s increase, OR = 1.021, 95% CI: 1.006–1.037), and blood flow velocity (per 1 mL/min increase, OR = 1.027, 95% CI: 1.009–1.046) were found to be protective factors.

**Conclusion:**

oXiris CRRT is associated with a better prognosis in the treatment of severe abdominal infections. APTT, PT, D-dimer, and blood flow velocity are associated with early off-machine during oXiris CRRT.

## 1 Introduction

Sepsis is a common and life-threatening condition in intensive care units, with bacterial infection as its most important cause. Intra-abdominal infection (IAI) is a frequent cause of sepsis (1, 2). Its etiology is complex, and the condition varies widely. Untreated IAI allows rapid bacterial and endotoxin translocation into the systemic circulation, triggering dysregulated host responses that lead to sepsis and multiple organ failure. This can increase mortality by 30–50% (3). In the face of severe abdominal infection, in addition to active treatment of the primary disease and targeted anti-infection, timely and effective blocking of the sepsis cascade caused by endotoxin and inflammatory mediators is also one of the important means to reduce mortality (4, 5). Previously, clinicians often chose polymyxin B fixed fiber cartridge (PMX-DHP) or continuous hemoperfusion therapy with high cutoff (HCO) membranes, which has many potential adverse risks, such as serious complications, such as allergic reactions and transient hypotension (6). The new hemofiltration filter (oXiris), modified on the base membrane of AN69, which originally had adsorption, gives it strong endotoxin adsorption and the ability to inhibit inflammatory mediators. As highlighted in a recent state-of-the-art review by Yessayan et al. (7), extracorporeal blood purification therapies (including oXiris) show promise in modulating the inflammatory cascade in sepsis, though optimal protocols remain under investigation. Because it has powerful clearance mechanisms such as convective diffusion and adsorption, the survival rate of patients with abdominal infection using oXiris hemofilter in some studies is higher than that predicted by the severity score [Simplified Acute Physiology Score II (SAPS II)], and stabilizes hemodynamics, reduces the use of vasoactive drugs and reduces Sequential Organ Failure Assessment (SOFA) score, and improves hyperlactatemia (8, 9). However, in oXiris CRRT treatment, due to various reasons such as catheter dysfunction, line coagulation, filter coagulation, and puncture site oozing, the machine could not be removed at the expected optimal off-machine time point, so that the treatment had to be terminated prematurely. This not only increases additional healthcare costs and the treatment effect, and risk of infection (10) for critically ill patients, but also increases our workload, such as preparation of patient and machine lines, priming of closed extracorporeal circuits, and repeated connection of patient vascular access. Ensuring the optimal timing of CRRT initiation and maintaining its smooth operation are key and challenging aspects of nursing care. At present, there are many studies on early off-machine of treatment plans caused by multiple factors of CRRT (11, 12), but what factors affect the optimal off-machine time point during the application of oXiris CRRT in the treatment of patients with severe abdominal infection needs to be further explored in depth. This study sought to evaluate the clinical impact of oXiris CRRT in severe abdominal infections and identify risk factors for premature treatment termination (early off-machine) during the application of oXiris CRRT, in order to effectively monitor the *acid–base balance*, *coagulation status*, and other *signs* of patients and machines in patients with severe abdominal infection treated with oXiris CRRT, so as to develop a personalized nursing program to provide the basis.

## 2 Subjects and methods

### 2.1 Study subjects

This study conducted a retrospective analysis of 49 patients diagnosed with severe intra-abdominal infections admitted to the Intensive Care Unit (ICU) of the First Affiliated Hospital of Fujian Medical University from October 2020 to October 2023. Patients were divided into two groups based on whether they received oXiris CRRT (13): 22 patients received conventional treatment (the conventional CRRT group), and 27 patients received oXiris treatment (the oXiris group). The conventional CRRT group (22 cases) received standard M100 filter treatment, while the oXiris group (27 cases) received oXiris filter treatment. The enrollment time of the two groups covers the whole study period (October 2020 to October 2023), and there is no intentional time grouping or selective enrollment. This study is a retrospective observational study that did not use propensity score matching or other matching methods. Patient grouping relied on clinical treatment decisions, with attending physicians selecting filter types based on patient condition (e.g., sepsis severity, coagulation status). The attending physicians selected filter types (oXiris vs. conventional) based on clinical judgment, primarily considering the patient’s severity of sepsis (e.g., Acute Physiology and Chronic Health Evaluation II (APACHE II) score and vasopressor requirement), coagulation status (e.g., risk of bleeding), and local resource availability. While specific criteria were not formally standardized, common practice favored oXiris for patients with suspected severe endotoxemia or refractory inflammatory responses, whereas conventional CRRT was used for standard renal support. This non-uniform allocation highlights the need for standardized treatment protocols in future research to minimize allocation bias. The study protocol was approved by the medical ethics committee of the First Affiliated Hospital of Fujian Medical University, China (No. [2015] 084–2). Written informed consent was waived by the institutional ethics committee (Approval No. [2015] 084–2) because the study involved a retrospective analysis of de-identified clinical data, in accordance with local regulations on observational research (Chinese Ministry of Health Ethics Regulations 2016).

### 2.2 Inclusion criteria

(1) Patients met the diagnostic criteria for severe intra-abdominal infection, confirmed by history, physical examination, and abdominal computed tomography (CT), demonstrating gastrointestinal perforation, necrosis, gangrene, or surgical complications; (2) Body temperature >37.3°C or <36.0°C, white blood cell count >10 × 10^9^/L or <4 × 10^9^/L; (3) Abdominal drainage fluid or diagnostic paracentesis bacterial smear, culture positive; Microbial etiology was determined via abdominal fluid culture, with common pathogens including *Escherichia coli* (68.4%), *Klebsiella pneumoniae* (15.3%), and anaerobic bacteria (16.3%). Antibiotic regimens followed local guidelines for severe intra-abdominal infections, with empirical coverage adjusted based on culture results. Comorbidities (e.g., diabetes mellitus and hypertension) were recorded and compared between groups (Table 1). (4) Patients diagnosed with sepsis, according to the sepsis criteria (Sepsis 3.0) defined by the European Society of Intensive Care Medicine (ESICM) and the Society of Critical Care Medicine (SCCM), requiring vasopressors to maintain mean arterial pressure ≥65 mm Hg (1 mm Hg = 0.133 kPa) after active fluid resuscitation, and arterial blood lactate >2 mmol/L.

**Table 1:** Comparison of clinical data and pretreatment indicators between the oXiris CRRT group and conventional treatment group.

Indicator	oXiris CRRT (*N* = 22)	Conventional CRRT (*N* = 27)	*p*
Gender (Male, *n*, %)	15 (77.3%)	55.6%	0.112
Age (years, mean ± SD)	54.68 ± 15.39	62.00 ± 21.36	0.182
BMI (Kg/m^2^, median (IQR))	23.40 (21.30, 29.92)	25.32 (21.56, 31.09)	0.553
HR (beat/min, median (IQR))	119 (102, 125)	111 (95, 120)	0.205
MAP (mmHg, median (IQR))	69.83 (60.65, 84.75)	76.00 (69.51, 87.00)	0.165
Lac (mmol/l, median (IQR))	5.95 (2.9, 9.6)	4.40 (3.5, 7.6)	0.725
PCT (ng/ml, median (IQR))	25.15 (3.56, 90.32)	26.39 (9.05, 56.22)	0.856
IL-6 (pg/ml, median (IQR))	1803 (709.28, 3329.50)	2013 (430.80, 3116.00)	0.658
Noradrenaline dose (μg/kg)/min, median (IQR)	0.74 (0.31, 1.26)	0.62 (0.25, 1.12)	0.637
APACHEII (mean ± SD)	25.77 ± 4.89	25.41 ± 3.77	0.769
SOFA (mean ± SD)	13.32 ± 3.40	14.22 ± 2.52	0.291

Body Mass Index refers to body mass index. MAP (mean arterial pressure) = (systolic blood pressure + 2 × diastolic blood pressure)/3. (μg/kg)/min: Dose per kilogram of body weight per minute.

Data are presented as mean ± standard deviation for normally distributed variables or median (interquartile range) for non-normally distributed variables, as assessed by the Shapiro-Wilk test.

Exclusion criteria: (1) patients who have received CRRT before ICU admission; (2) Patients with hematological malignancies; (3) Patients with new coronavirus pneumonia; (4) Pregnant women; (5) Minors (<18 years old) meeting one of the criteria can be excluded.

Baseline comparability verification: the baseline data of sex, age, and body mass index (BMI), heart rate (HR) before treatment, mean arterial pressure (MAP), blood lactic acid (LAC), procalcitonin (PCT), interleukin-6 (IL-6), norepinephrine dose, APACHE II score and SOFA score were analyzed using *t*-test or chi-squared test, and there was no statistical difference (*p* > 0.05, Table 1), indicating that the two groups were comparable.

### 2.3 Data collection

#### 2.3.1 General treatment

After transfer to the intensive care unit (ICU), patients in both groups received active treatment according to international guidelines for the treatment of sepsis and septic shock (14), including treatment of primary disease, active infection control, fluid resuscitation, collection of multiple pus samples for examination, and support of multiple organ functions. In clinical practice, in addition to preparing for CRRT on the machine to ensure the quality of equipment and consumables, the correctness of the puncture site and the absence of oozing exudate, the dose of drugs and replacement solutions within the validity period, and the safety and hygiene of the treatment environment. It is necessary to closely monitor the changes in vital signs and disease conditions of patients; regularly inspect whether the patient pipeline is unobstructed, whether the machine operates normally, reduce the occurrence of blood leakage, etc.; periodically pay attention to the fluid balance of patients, timely adjust the infusion volume and speed, to avoid excessive dehydration or fluid retention.

#### 2.3.2 CRRT filters

##### 2.3.2.1 oXiris group

In addition to general treatment, patients started receiving blood purification treatment within 24 h after admission to the ICU. Central venous catheterization was performed, femoral venous indwelling catheter (11.5F) was selected, Prisma-flex blood purification machine was used, AN69-oXiris filter (Baxter, United States) was selected for the filter, the treatment mode was continuous venovenous hemofiltration (CVVH), the therapeutic dose was 30–40 mL/(kg·h), the blood flow was 120–180 mL/h, and heparin anticoagulation or no heparin treatment was selected according to the patient’s coagulation function and bleeding and liver function.

##### 2.3.2.2 Conventional CRRT group

In addition to general treatment, patients start receiving blood purification treatment within 24 h after admission to the ICU. Venous catheterization was performed. The femoral venous indwelling catheter (11.5F) was selected. Prisma-flex blood purification machine: Manufactured by Baxter International Inc., Deerfield, IL, United States. M100 filter: Gambro Lundia AB, Lund, Sweden. Continuous venovenous filtration (CVVH) was used as the treatment mode. The therapeutic dose was 30–40 mL/(kg·h), and blood flow was 120–180 mL/h. Heparin anticoagulation or heparin-free treatment was selected according to the patient’s coagulation function, presence or absence of active bleeding, and liver function.

#### 2.3.3 Other data/covariates

Heart rate (HR), mean arterial pressure (MAP), blood lactate level (LAC), serum procalcitonin (PCT), interleukin-6 (IL-6), norepinephrine dosage (μg/kg/min), Acute Physiology and Chronic Health Evaluation II (APACHE II), Quick Sequential Organ Failure Score (qSOFA), length of hospital follow-up, 28-day mortality were collected before and 72 h after starting CRRT in both groups.

#### 2.3.4 Recorded items

Included general patient information, the total running time of oXiris CRRT, number of sessions, blood flow rates during CRRT, anticoagulation methods, predilution ratios, and whether blood products were used. Laboratory parameters measured before each oXiris CRRT session included hemoglobin, hematocrit, platelet count, activated partial thromboplastin time, prothrombin time, international normalized ratio, fibrinogen, and D-dimer.

### 2.4 Outcome measures

(1) The treatment effect in this study refers to: 72 h after CRRT treatment, respiratory rate, HR, lactate (Lac) level, IL-6, noradrenaline dose (μg/kg/min), APACHE-II, SOFA were improved (*p* < 0.05) and the cumulative survival rate was compared between the two groups.

(2) Indications for relevant risk factors of early off-machine: the termination of treatment due to failure to complete the target dehydration or failure to continue 72 h of CRRT is defined as the relevant risk factors of non-early off-machine of CRRT, and vice versa is called the optimal off-machine point of CRRT (15). Specifically: transmembrane pressure (TMP) > 250 mm Hg (1 mm Hg = 0.133 kPa), filter coagulation Grade II or above, various alarms cannot be ruled out, leading to machine stoppage. Filter clotting was based on (16) Grade III clotting. Grade 0: no coagulation or several fibers coagulation; Grade I: <10% fibers coagulation; Grade II: <50% fibers coagulation; Grade III: >50% fibers coagulation.

### 2.5 Statistical methods

Statistical analysis was conducted using the Statistical Package for Social Sciences (SPSS) version 26.0 software: IBM Corp., Armonk, NY, United States. Count data were expressed as frequency and percentage, while measurement data were presented as mean ± standard deviation (normally distributed) or median (interquartile range [IQR]) (non-normally distributed). For continuous variables, normality was assessed using the Shapiro–Wilk test. Parametric tests (*t*-tests) were used for normally distributed data, while non-parametric tests (Mann–Whitney *U* test) were applied to non-normally distributed variables (e.g., lactate and PCT). Logistic regression equations were employed to construct predictive models and generate forest plots. Survival rates were analyzed using the Kaplan–Meier method, and the area under the receiver operating characteristic (ROC) curve was used to assess model predictive performance. The Hosmer–Lemeshow test was applied to evaluate model consistency. The significance level was set at *α* = 0.05. Anticoagulation methods (heparin vs. heparin-free) and predilution ratios were not included in the multivariate model due to limited sample size and imbalance (heparin-free group: *n* = 30), which precluded robust subgroup analysis. Future studies with larger samples should explore these factors. The sample size was determined by clinical case availability during the study period, without prior power calculation. Given the low incidence of severe abdominal infection requiring CRRT and limited early clinical use of oXiris filters, accumulating a larger sample was challenging in a single center.

## 3 Results

### 3.1 General data of study subjects

Thirty-two male and 17 female patients, aged 25–96 years, with sepsis due to abdominal infection, were enrolled. Among them, 22 patients received oXiris CRRT and 151 patients received CRRT. There were 54 cases (35.7%) of CRRT due to achieving the purpose of treatment or treatment time; 3 cases (2.0%) of CRRT due to giving up treatment and death; 6 cases (4.0%) of CRRT due to treatment-related factors such as going out CT examination; 88 cases (58.3%) of CRRT due to risk factors related to filter coagulation grade II or above, machine prompted line coagulation and various alarms that could not be excluded. Among them, two cases of hemofilter failure were forced to get off the machine (1.3%), and 1 case of filter rupture (0.6%).

### 3.2 Comparison of clinical treatment and therapeutic effect of different filters

**Figure 1:**
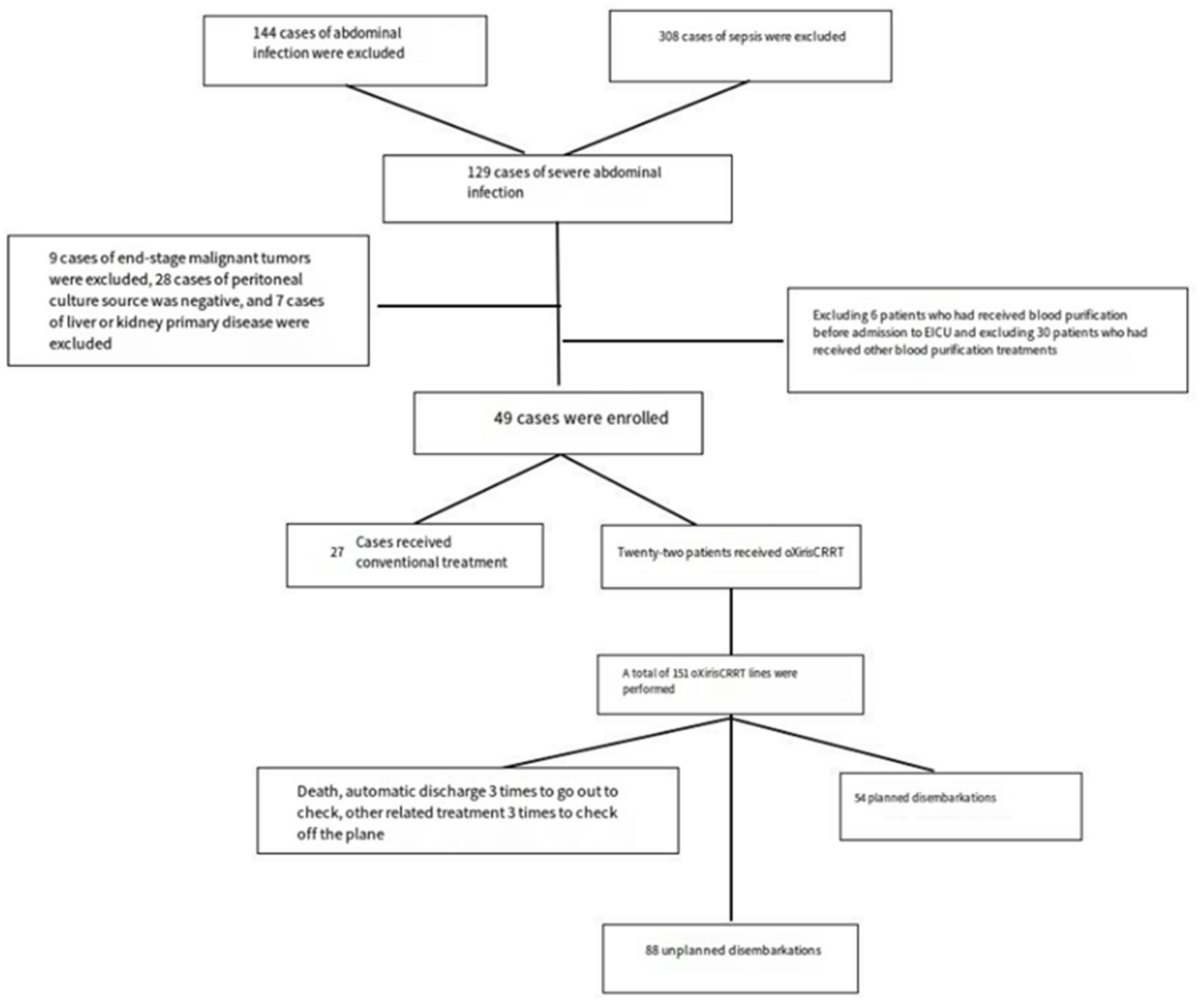
Flowchart.

As shown in Table 1, there was no significant statistical difference in sex, age, and BMI between the oXiris group and the conventional treatment group (*p* > 0.05); and there were no statistical differences in HR, MAP, Lac, PCT, IL-6, SOFA, noradrenaline dose [(μg/kg)/min], APACHE II and other indicators before the start of treatment (*p* > 0.05) (Figure 1).

As shown in Table 2, comparing the changes of indicators at 72 h between the two groups, except MAP and PCT, HR, Lac, IL-6, noradrenaline dose, APACHE-II, and SOFA were improved (*p* < 0.05).

**Table 2:** Comparison of data between the oXiris CRRT group and conventional treatment group after 72 hours of treatment.

**Indicator**	**oXiris CRRT (22)**	**Conventional CRRT (27)**	*p*
HR (beat/min, mean ± SD)	92.68 ± 16.46	104.30 ± 15.67	0.017
MAP (mmHg, mean ± SD)	83.87 ± 12.79	76.24 ± 15.15	0.067
Lac (mmol/l, median (IQR))	1.85 (1.30, 4.83)	5.10 (2.3, 7.6)	0.021
PCT (ng/ml, median (IQR))	8.68 (1.07, 17.33)	18.63 (4.93, 53.40)	0.074
IL-6 (pg/ml, median (IQR))	320.50 (126.75, 463.50)	1055.00 (200.40, 1987.00)	0.009
Noradrenaline Dose (μg/kg/min, median (IQR))	0.19 (0.02, 0.85)	0.73 (0.15, 1.26)	0.035
APACHEII (mean ± SD)	18.55 ± 5.83	23.96 ± 5.33	0.001
SOFA (mean ± SD)	1064 ± 3.37	14.00 ± 3.19	<0.001

Data are presented as mean ± standard deviation for normally distributed variables or median (interquartile range) for non-normally distributed variables, as assessed by the *Shapiro-Wilk* test.

Significant differences are denoted by *p* < 0.05.

**Figure 2:**
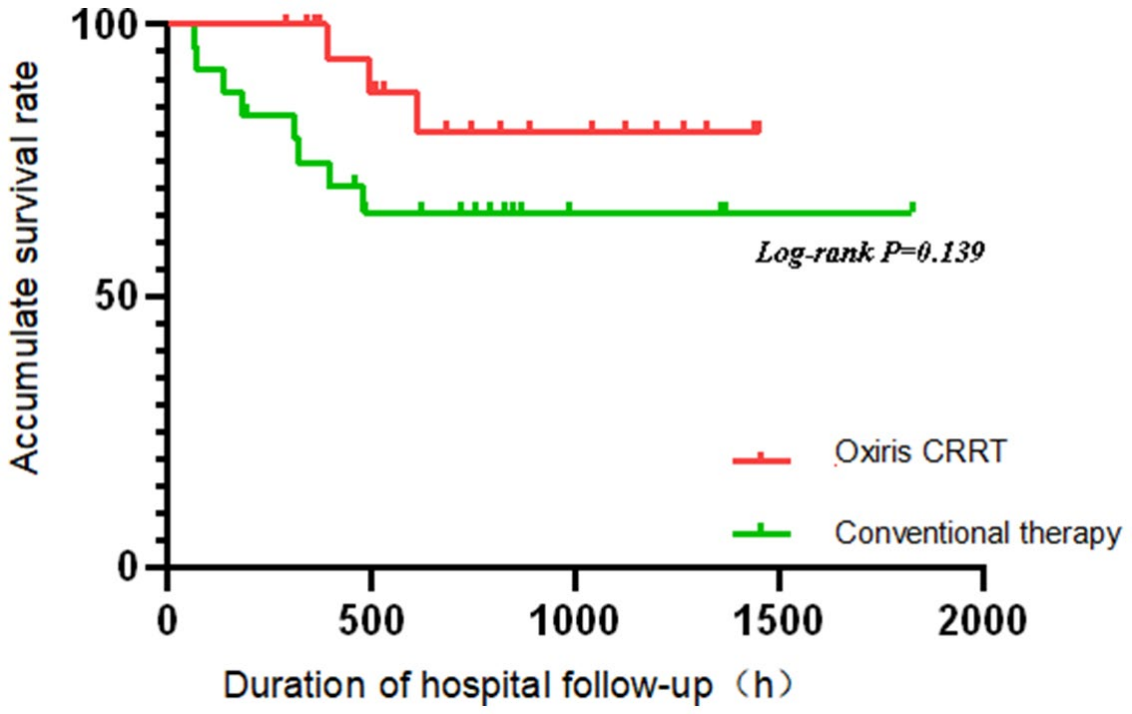
oXiris CRRT in-hospital survival analysis.

The Kaplan–Meier method was used to analyze the prognosis of patients with severe abdominal infection in the oXiris CRRT group and the conventional treatment group. As can be seen from Figure 2, the survival rate decreased faster in the conventional treatment group than in the oXiris CRRT group over time; however, the log-rank test revealed that there was no statistically significant difference in survival between the two groups of patients (*p* = 0.139). The non-significant survival difference (*p* = 0.139) is likely due to the small sample size (*n* = 49), which had limited power to detect subtle survival benefits. The observed trend (faster survival decline in the conventional group) suggests a potential clinical benefit trend. However, the non-significant survival difference (*p* = 0.139) highlights the need for larger trials to confirm this hypothesis-generating finding.

### 3.3 Analysis of influencing factors of risk factors associated with early off-machine of oXiris CRRT

#### 3.3.1 Definition clarification

Early off-machine was defined as premature termination of CRRT before 72 h due to technical failures (e.g., filter coagulation ≥ Grade II, line clotting, unresolvable alarms) or failure to achieve target fluid removal. In contrast, non-early off-machine referred to planned termination after completing 72-h treatment or meeting clinical endpoints (e.g., improved organ function).

#### 3.3.2 Distribution of off-machine causes

Among 142 CRRT sessions, 88 cases (58.3%) underwent early off-machine, primarily due to technical complications:

1. Filter coagulation (Grades II and III): 67 cases (76.1%, defined as ≥50% fiber clotting);
2. Line clotting or unresolvable alarms: 21 cases (23.9%).

The remaining 54 cases (35.7%) achieved non-early off-machine by completing 72-h treatment or resolving clinical indications (e.g., lactate ≤ 2 mmol/L, vasopressor-free hemodynamics).

#### 3.3.3 Univariate analysis of risk factors

To clarify the association between variables and early off-machine risk, we categorized factors as follows (Table 3):

**Table 3:** Results of univariate analysis of combined oXiris CRRT for early termination of disembarkation plan in severe abdominal infection.

**Factor**	Optimal off-machine time (*n* = 54)	Early off-machine (*n* = 88)	*p*
Anticoagulation method (heparin, n, %)	42 (37.5%)	70 (72.5%)	0.802
Predilution (%): 0/25/50/75/100	2 (33.3%)	4 (66.7%)	0.971
	14 (38.9%)	22 (61.1%)	
	14 (40.0%)	21 (60.0%)	
	17 (39.5%)	26 (60.5%)	
	7 (31.8%)	15 (68.2%)	
Number of blood products used(median (IQR))	1 (0, 2.25)	1 (0, 2.00)	0.981
Blood flow rate (ml/min, median (IQR))	160 (150, 180)	150 (122.5, 177.5)	0.002
Hemoglobin (g/L, mean ± SD)	83.75 ± 22.23	85.24 ± 20.89	0.694
Hematocrit (%, median (IQR))	31.3 (28.00, 36.33)	31.7 (28.53, 36.60)	0.393
Platelets (109/L, mean ± SD)	95.54 ± 30.17	93.02 ± 30.10	0.630
PT (s, median (IQR))	18.9 (15.60, 22.25)	16.7 (14.30, 20.43)	0.037
INR(median (IQR))	1.1 (0.98, 1.33)	1.15 (0.90, 1.40)	0.676
APTT (s, median (IQR))	91.0 (79.0, 115.0)	82.0 (71.0, 102.75)	0.012
Fibrinogen (mg/L, median (IQR))	3.4 (2.50, 5.10)	3.4 (2.43, 4.90)	0.822
D-D (ng/mL, median (IQR))	5.6 (2.30, 8.80)	8.65 (4.20, 12.98)	0.008

##### 3.3.3.1 Factors associated with reduced early off-machine risk

Higher values of the following indicators were linked to a lower probability of premature CRRT termination:

(1) Blood flow velocity: Each 1 mL/min increase was associated with a 2.7% higher odds of completing treatment (odds ratio (OR) = 1.027, 95% confidence interval (CI): 1.009–1.046, *p* = 0.002), indicating that faster flow rates mitigate filter clotting.

(2) Prolonged coagulation times:

PT: Each 1-s prolongation increased treatment completion odds by 11.7% (OR = 1.117, 95% CI: 1.017–1.226, *p* = 0.037).

APTT: Each 1-s prolongation increased completion odds by 2.1% (OR = 1.021, 95% CI: 1.006–1.037, *p* = 0.012).

Rationale: Prolonged PT/APTT reflects reduced coagulability, decreasing extracorporeal circuit clotting risk.

##### 3.3.3.2 Factor associated with increased early off-machine risk

Higher D-dimer levels were associated with premature termination:

Each 1 ng/mL increase was linked to 7% lower odds of completing treatment (OR = 0.930, 95% CI: 0.866–0.999, *p* = 0.008), indicating that elevated D-dimer (a marker of hypercoagulability) promotes filter clotting.

#### 3.3.4 Multivariate prediction model

The final model integrated prothrombin time (PT), activated partial thromboplastin time (APTT), blood flow velocity, and D-dimer: (1) Formula: Logit(p) = −6.791 + 0.09 × PT + 0.018 × APTT + 0.023 × flow velocity − 0.078 × D-dimer. (2) Performance: ROC AUC = 0.741 (95% CI: 0.659–0.822), sensitivity = 94.4%, specificity = 45.5%. Indicating moderation accuracy in distinguishing technical failure related early off machine from planned termination (Table 4).

**Table 4:** Multivariate logistic analysis of risk factors associated with early off-machine during oXiris CRRT.

**Variable**	**Beta**	**SE**	**Waldc** ^**2**^	*p*	**OR**	**95% *CI***
Constant	−6.791	1.834	13.704	0.000	0.001	— —
PT (s)	0.090	0.043	4.450	0.035	1.094	1.006-1.190
APTT (s)	0.018	0.007	5.990	0.014	1.018	1.004-1.033
Blood flow velocity (ml/min)	0.023	0.009	7.302	0.007	1.023	1.006-1.041
D-D (ng/mL)	-0.078	0.035	4.936	0.026	0.925	0.863-0.991

SE, standard error.

### 3.4 Construction and efficacy evaluation of the prediction model of risk factors associated with oXiris CRRT early removal

Comprehensive analysis of various influencing factors of oXiris CRRT during the treatment of severe abdominal infections: the optimal off-machine time prediction model was: Logit(P) = −6.791 + 0.09 × PT + 0.018 × APTT + 0.023 × blood flow velocity − 0.078 × D-dimer. The ROC curve showed an AUC of 0.741 (95% CI: 0.659–0.822), with a maximum Youden index of 0.399, optimal cutoff value of 0.260, sensitivity of 0.944, and specificity of 0.455. The model showed a good fit according to the Hosmer–Lemeshow test (*c*^2^ = 7.767, *p* = 0.457). The low specificity (45.5%) may reflect the complex nature of early off-machine, which is influenced by multiple unmeasured factors (e.g., catheter dysfunction, operator experience). The model’s high sensitivity (94.4%) prioritizes identifying patients at risk of early off-machine, which can guide clinical vigilance despite false positives.

## 4 Discussion

The CRRT primarily reduces inflammatory mediators, removes metabolic wastes, stabilizes immune balance, and enhances vascular endothelial function to minimize organ damage (17). The oXiris filter, an upgrade of AN69, retains strong inflammatory adsorption and matches the efficiency of polymyxin adsorption columns (18). It can be used for venovenous hemofiltration (CVVH) or continuous venovenous hemodiafiltration (CVVHDF) to correct blood circulation, stabilize hemodynamics, and improve organ functions. Uniquely, the oXiris filter offers renal replacement therapy while removing endotoxins and adsorbing cytokines.

Intra-abdominal infections (IAIs) often involve mixed infections (19), with *E. coli* being the most common pathogen and a major source of endotoxin, significantly contributing to sepsis. According to Honore et al. (20), endotoxins from Gram-negative infections and those released due to gut dysbiosis after Gram-positive infections both exacerbate sepsis. Endotoxins in the bloodstream trigger immune and coagulation responses, leading to sepsis. This study shows that elevated levels of interleukin-6 (IL-6), PCT, and other inflammatory markers indicate that endotoxin-induced immune overactivation primarily causes multiple organ dysfunction. Thus, reducing toxin levels in patients is.

Procalcitonin (PCT) (21–23) is used to assess sepsis severity and guide antibiotic use (24, 25), as it is regulated by cytokines and endotoxin (26), and correlates with patient mortality (27). IL-6, produced by T cells, also relates to mortality by enhancing B cell antibody function (28). Table 2 shows significant improvements in PCT and IL-6 levels in patients treated with oXiris CRRT for 72 h, likely due to effective endotoxin clearance by the oXiris filter, preventing immune system imbalance, as supported by recent clinical studies. The oXiris filter’s endotoxin adsorption capacity is inferred from improvements in PCT (8.68 vs. 18.63 ng/mL, *p* = 0.074) and IL-6 (320.50 vs. 1,055.00 pg./mL, *p* = 0.009) (Table 2). These markers are tightly linked to endotoxin activity: PCT is upregulated by endotoxin via Toll-like receptor four signaling (26), while IL-6 reflects the downstream inflammatory cascade triggered by endotoxin (28). Although direct endotoxin measurement was unavailable, these surrogate markers provide indirect evidence of oXiris-mediated endotoxin clearance. Patients treated with the oXiris filter exhibited more stable vital signs, with a greater decrease in heart rate and improved hemodynamics (notably lower lactate levels and norepinephrine doses) compared to the control group (*p* < 0.05). Organ function also significantly improved, as indicated by reduced APACHE II and SOFA scores (*p* < 0.05). However, there was no significant difference in survival rates between groups (*p* > 0.05), aligning with findings from Navas et al. (29). Some studies suggest oXiris may reduce mortality (29–31), but in this study, while the KM survival curve showed no statistical difference, oXiris-induced improvements in hemodynamics and inflammatory markers suggest potential clinical benefit, which requires confirmation in large-scale studies. The term ‘better prognosis’ in the original manuscript referred to improvements in physiological parameters (e.g., lactate clearance and SOFA score reduction), which are established surrogate markers of clinical benefit. Although survival differences were non-significant (*p* = 0.139), the observed trends align with prior studies (31) and suggest potential long-term benefits requiring confirmation in large-scale randomized controlled trials (RCTs). Survival outcomes were influenced by factors such as tube replacement frequency, severity of abdominal infection, nutritional status, and drainage conditions. While several studies suggest mortality benefits with oXiris CRRT (29–31), this study’s non-significant survival trend highlights methodological heterogeneity in the literature. For example, Navas et al. (29) used a case–control design with polymyxin B hemoperfusion, whereas Wang et al. (31) conducted a meta-analysis of retrospective studies. Such variability in study design and patient populations underscores the need for standardized, prospective trials to reconcile these findings.

**Figure 3:**
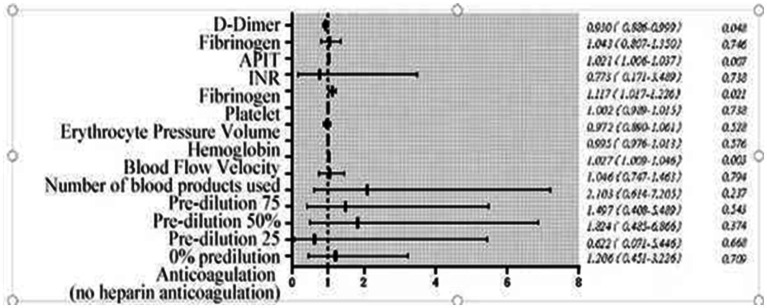
Forest plot of optimal off-machine time.

**Figure 4:**
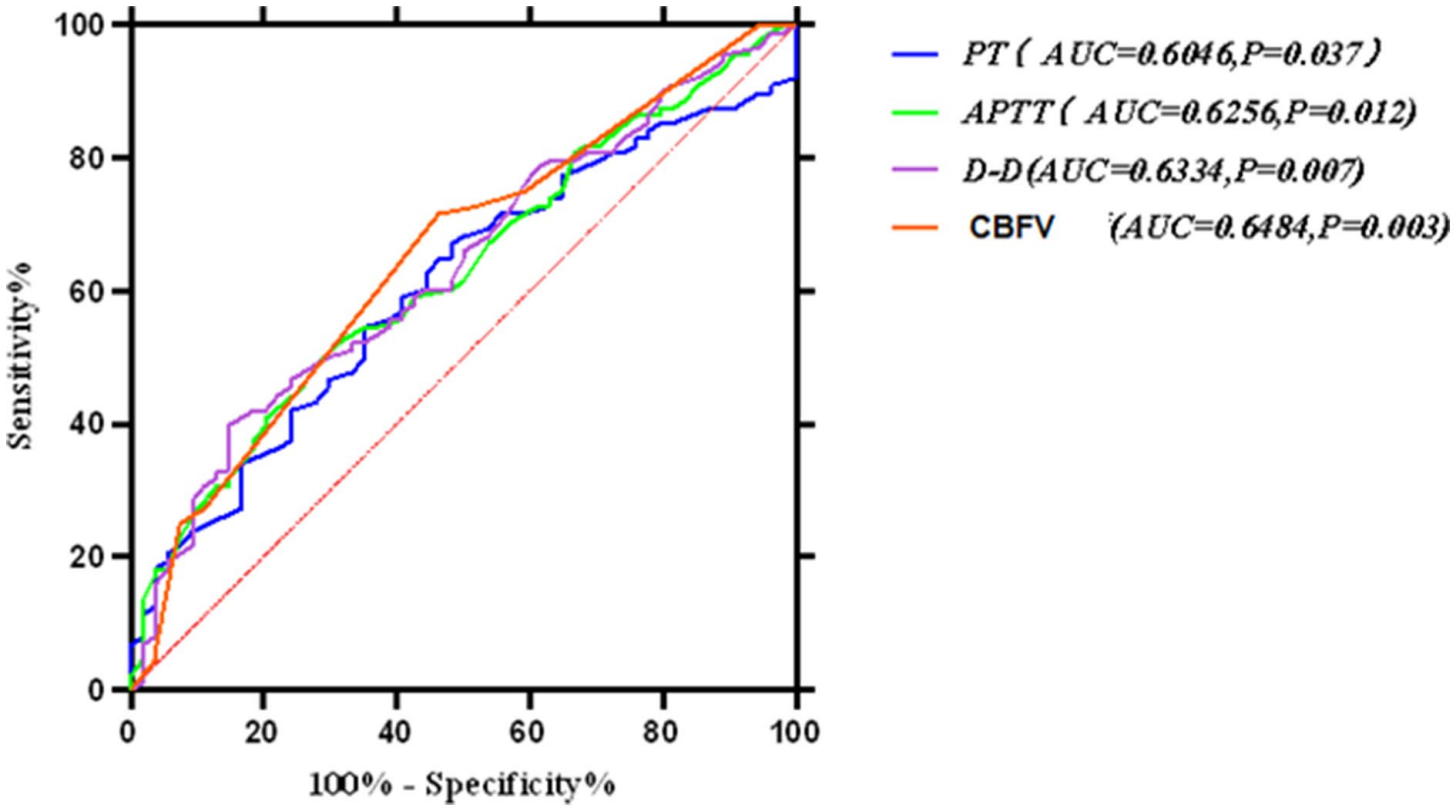
Receiver operating characteristic (ROC) curve for the multivariate prediction model of early off-machine risk. The model demonstrated an area under the curve (AUC) of 0.741 (95% CI: 0.659–0.822), with an optimal cutoff value of 0.260 (sensitivity = 94.4%, specificity = 45.5%).

**Figure 5:**
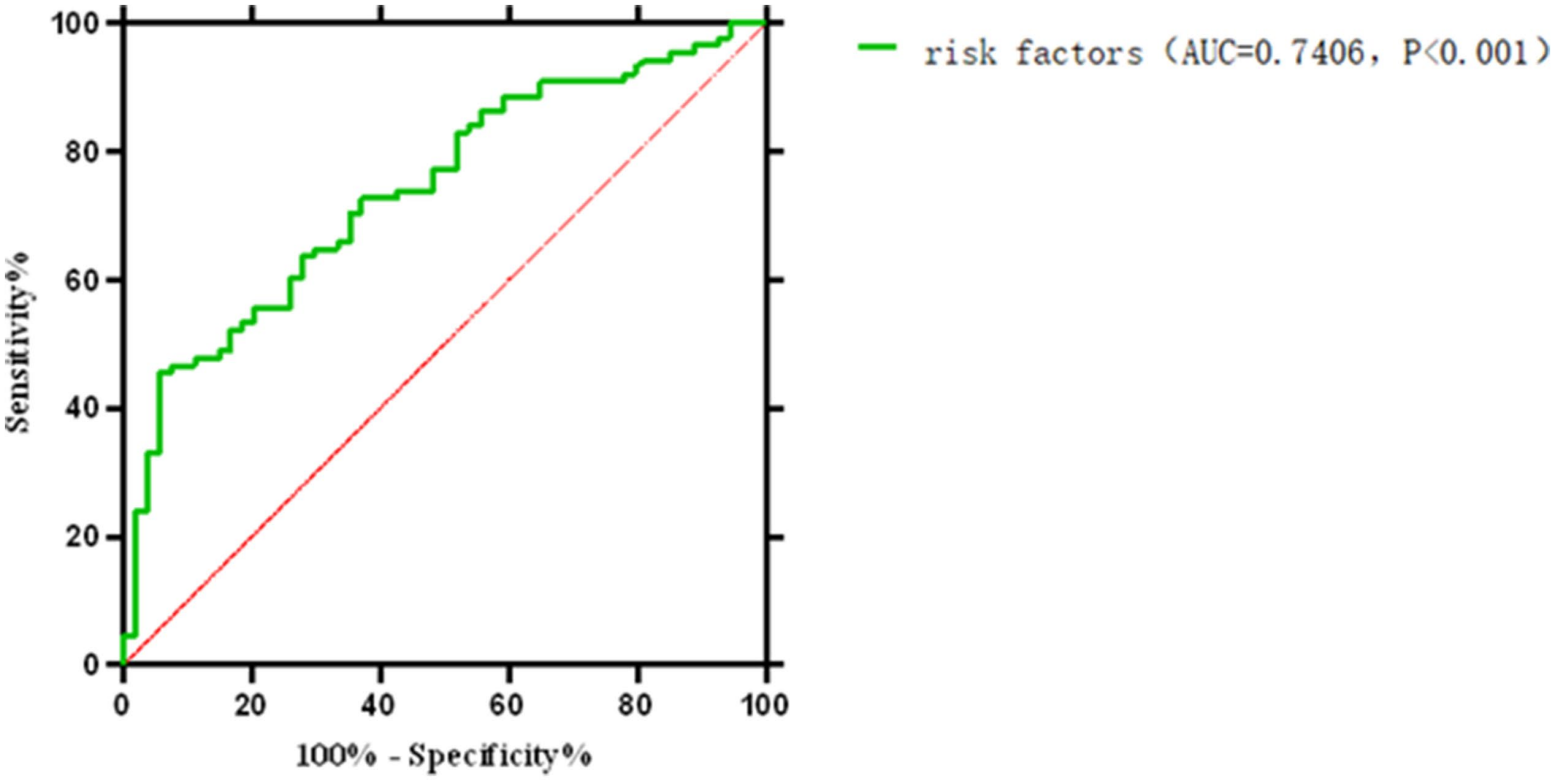
ROC curve for the comprehensive prediction of risk factors associated with early off-machine of oXiris CRR.

Subgroup analyses of anticoagulation methods (heparin vs. heparin-free) were underpowered due to small sample sizes, so we focused on the main effects of coagulation markers and blood flow velocity. Ending CRRT prematurely reduces the clearance of endotoxins and inflammatory mediators, causes blood loss, raises treatment costs, and increases our workload. This study identified risk factors for not ending treatment early, primarily failing to meet the target fluid loss or complete 72 h of continuous treatment. Figure 3 and Table 3 show that blood flow velocity during oXiris CRRT, along with initial APTT, PT, and D-dimer levels, are related to these risk factors. Figures 4, 5 illustrate the risk factors of using oXiris CRRT for severe abdominal infections. By actively managing these factors—such as extending APTT, increasing blood flow velocity, and adjusting acid–base balance—we can reduce early treatment termination, lower family costs, decrease patient reinfection risk, and lighten our workload.

Prolonged APTT indicates abnormalities in coagulation factors or the presence of anticoagulants, while shortened APTT suggests a hypercoagulable state. Some studies show that increased APTT is linked to a lower risk of circuit clotting (32) and longer CRRT filter life (33). Prothrombin time (PT) is a key indicator of coagulation function; a shortened PT signifies a hypercoagulable state, increasing the risk of thrombus formation and pipeline blockage (34). Prolonged PT and APTT reflect reduced coagulability, suggesting that maintaining therapeutic anticoagulation within target ranges may mitigate early off-machine. Increasing blood flow velocity ≥160 mL/min could also reduce filter clotting risk, as observed in the risk factor group. CRRT blood flow velocity is a risk factor for early off-machine oXiris CRRT; slower blood flow increases the likelihood of coagulation and premature disconnection. In this study, the median blood flow velocity was 160 mL/min in the risk factor group and 150 mL/min in the early off-machine group, with a statistically significant difference (*p* < 0.05), corroborating findings from other studies (35, 36). However, the factors influencing early off-machine risks are complex and cannot be solely explained by traditional coagulation markers like PT, APTT, D-dimer, or blood flow velocity (37, 38). A recent meta-analysis identified various factors affecting filter life, including catheter access, circuit, and patient-related factors (39). Therefore, we need to include more comprehensive indicators to accurately maintain the multifactor risk calculation formula for related risk factors of oXiris CRRT early removal, to reduce unnecessary medical resource waste, such as tubing and filters.

In this study, various factors were individually used to develop a multivariate risk formula for predicting early off-machine risk factors, as shown in Figure 4. Typically, an ROC curve area of 0.5–0.7 indicates low prediction accuracy, 0.7–0.9 indicates moderate accuracy, and >0.9 indicates high accuracy (40). We then combined these factors and applied the Hosmer–Lemeshow test to assess the formula’s fit, finding no significant differences, which suggests that the formula’s predictions align well with actual incidence rates. The model’s ROC curve area was 0.741, with a Youden index of 0.399 and an optimal diagnostic value of 0.260. It showed a sensitivity of 0.944 and specificity of 0.455, indicating its effectiveness in predicting and differentiating risk factors for early off-machine in oXiris CRRT during severe abdominal infection treatment. A model score of ≥0.260 suggests a high likelihood of achieving the optimal off-board time plan. Scores below this threshold require clinician consultation to adjust treatment parameters. Scores near the critical value also necessitate timely reporting to develop a prospective intervention plan, such as adjusting anticoagulant dose and blood flow rate, to ensure optimal outcomes.

Sepsis is primarily characterized by a systemic inflammatory response syndrome (SIRS) triggered by endotoxins and inflammatory factors (1, 4). The oXiris filter helps correct immune imbalances by adsorbing endotoxins and inflammatory mediators such as IL-6 and TNF-*α* (9). This mechanism is relevant in infections such as abdominal infections, pneumonia, and bloodstream infections. Yessayan et al. (7) noted that extracorporeal blood purification therapy, including oXiris, can modulate the inflammatory cascade in various sepsis types, suggesting its potential to improve hemodynamics and reduce inflammation in other infections. Abdominal infections often involve a compromised intestinal barrier, allowing direct endotoxin entry into the bloodstream, frequently resulting in mixed infections (e.g., *E. coli* and anaerobic bacteria) (19), which may alter endotoxin load and coagulation compared to other infections (such as lung infection with single bacteria). The study identifies Early Off-Machine risk factors (e.g., D-dimer, Pt, APTT) linked to coagulation function, which may differ from coagulation patterns in other infections. For instance, sepsis from lung infections might rely more on local inflammatory factors than endotoxin activation of coagulation (28). Thus, the study’s risk factor model may need validation for other infections.

Sepsis is a highly varied syndrome with significant differences in pathophysiology, clinical presentation, and treatment response (1). Yang et al. (41) used cluster analysis to categorize post-laparoscopic surgery sepsis patients into four subphenotypes, revealing notable differences in age, coagulation, and inflammation among these groups. This highlights the importance of considering sepsis heterogeneity in treatment strategies. The study also suggests that while oXiris CRRT can effectively adsorb endotoxins and inflammatory factors, its efficacy may vary across different patient subgroups. The study did not conduct a prior subgroup analysis. Still, baseline data indicated that IL-6 and blood lactic acid levels improved more in the oXiris group after 72 h, although survival differences were not statistically significant (*p* = 0.139). This could be due to sepsis heterogeneity; patients with impaired coagulation might benefit more from oXiris’ anticoagulation and inflammation regulation (OR = 1.117, 95% CI: 1.017–1.226), while those with normal coagulation may respond less (2). Additionally, early weaning risk factors like D-dimer and blood flow velocity suggest varying treatment tolerance across subgroups.

This study has several limitations: (1) Retrospective design lacks direct comparison with conventional CRRT, potentially limiting full demonstration of oXiris efficacy (9, 31). (2) The absence of direct endotoxin measurement is a key limitation. While PCT and IL-6 changes suggest endotoxin clearance, future studies should use the LAL assay to quantify plasma endotoxin levels and validate the mechanistic link between oXiris and endotoxin adsorption (7). This study did not explicitly control for microbial etiology or specific antibiotic regimens in the multivariate model, which may introduce residual bias. For example, patients with *E. coli*-dominant infections might respond differently to oXiris due to endotoxin load. Future studies should incorporate these variables to refine risk prediction (31, 42). Additionally, the multivariate model did not adjust for microbial etiology (e.g., *E. coli* vs. anaerobic infections) or specific antibiotic regimens, which may influence both inflammatory responses and treatment outcomes. For instance, patients with Gram-negative infections (the majority in this cohort) might have higher endotoxin loads, potentially confounding the association between oXiris use and clinical improvement. Future research should stratify by pathogen type and incorporate antibiotic exposure into risk models to refine these findings. (3) In this study, under the condition of a single center, the proportion of cases requiring CRRT due to severe abdominal infection complicated with sepsis is low, and the early clinical application rate of the oXiris filter is low, which makes it difficult to accumulate large samples in the short term. Small single-center sample (*n* = 49, oXiris = 27) aligns with similar studies (9, 31), mitigated by multivariate regression (Table 4) and survival analysis. Subgroup analyses of anticoagulation strategies were underpowered (heparin group: *n* = 112, heparin-free group: *n* = 30), precluding robust conclusions about optimal anticoagulation in oXiris CRRT. Notably, the majority of oXiris-related evidence cited here (9, 31) originates from retrospective studies or case series, which carry inherent biases. This limitation emphasizes the urgent need for prospective, randomized controlled trials to establish definitive efficacy and optimal treatment protocols for oXiris CRRT in sepsis. The small single-center sample size (*n* = 49) inherently limits statistical power; larger multicenter trials are needed to validate the observed associations between oXiris use and clinical outcomes. (4) Nursing variables (e.g., treatment timing, anticoagulant dose) require validation in RCTs. (5) Single-center bias is reduced by baseline comparability (Table 1) and multivariate correction. Still, future studies should adopt Yang et al.’s (42) target test simulation (TTE) framework—a method for simulating RCT conditions in observational data—to control confounding factors like infection source management and nutritional support.

In summary, oXiris CRRT improved physiological markers (lactate, IL-6, and organ function scores) in severe abdominal infections, and APTT, PT, D-dimer, and blood flow velocity were associated with early off-machine. These findings provide insights for clinical monitoring and risk stratification during oXiris CRRT treatment. Combining the model with clinical judgment and real-time monitoring may improve specificity, which warrants exploration in future studies.

## Data Availability

The original contributions presented in the study are included in the article/supplementary material, further inquiries can be directed to the corresponding authors.
